# *Tae-miR396b* regulates *TaGRFs* in spikes of three wheat spike mutants

**DOI:** 10.7717/peerj.18550

**Published:** 2024-11-22

**Authors:** Ziping Yao, Qi Wang, Ying Xue, Zhiheng Liang, Yongjing Ni, Yumei Jiang, Peipei Zhang, Ting Wang, Qiaoyun Li, Lei Li, Jishan Niu

**Affiliations:** 1 National Centre of Engineering and Technological Research for Wheat, Henan Agricultural University, Zhengzhou, Henan, China; 2 Henan Engineering Research Center of Wheat Spring Freeze Injury Identification, Shangqiu Academy of Agricultural and Forestry Sciences, Shangqiu, China

**Keywords:** Wheat (*Triticum aestivum* L.), Spike, Mutant, Tae-miR396b, Growth regulating factor (GRF), Regulation

## Abstract

Tillering and spike differentiation are key agronomic traits for wheat (*Triticum aestivum* L.) production. Numerous studies have shown that miR396 and growth-regulating factor genes (*GRFs*) are involved in growth and development of different plant organs. Previously, we have reported that wheat miR396b (*tae-miR396b*) and their targets *TaGRFs* (*T. aestivum GRFs*) play important roles in regulating wheat tillering. This study was to investigate the regulatory roles of *tae-miR396b* and *TaGRFs* played during wheat spike development. Wheat cultivar Guomai 301 (wild type, WT) and its three sipke mutants dwarf round spike mutant (*drs*), apical spikelet sterility mutant (*ass*) and prematurely terminated spike differentiation mutant (*ptsd1*) were studied. Three homeologous genes of *tae-miR396b* on the long arms of chromosomes 6A, 6B, and 6D were identified, and they encoded the same mature miRNA. Complementary sequences of mature *tae*-*miR396b* were identified in 23 *TaGRFs*, indicating they were the target genes of *tae*-*miR396b*. *Tae-miR396b* had different regulatory effects on *TaGRFs* between Guomai 301 and its mutants. *TaGRF2-7A* was confirmed to be the target gene of *tae-miR396b* by molecular interaction assay. The expression levels of *tae-miR396b* and *TaGRFs* were different between WT and mutants *drs*, *ass* and *ptsd1* at the floret primordium visible (S1), the two awns/spikelet reaching apical meristem of the spikelet (S2), and the green anther stage (S3). The expression level of *tae-miR396b* in WT was significantly higher than that in mutants *drs* and *ass*. The most *TaGRFs* were negatively regulated by *tae-miR396b*. The abnormal expressions of *TaGRF1* (*6A*, *6D*), *TaGRF2* (*7A*, *7B*, *7D*), *TaGRF4* (*6A*, *6B*), *TaGRF5* (*4A*, *7A*, *7D*), and *TaGRF10* (*6A*, *6B*, *6D*) were important causes for abnormal spike development in the three mutants. This study laid foundation for further elucidating functions of *tae-miR396b* and *TaGRFs* underlying wheat spike development. Regulating *tae-miR396b* and *TaGRFs* will be a new approach for wheat high yield breeding.

## Introduction

Wheat (*Triticum aestivum* L.) is one of the most important crops in the world. Tillering and spike differentiation are two determining traits for wheat production, so they are of significant scientific and economic interest (Sreenivasulu & Schnurbusch, 2012). Per plant yield of wheat consists of fertile spikes per plant, fertile spikelets per spike, grains per spike, and grain weight (Sreenivasulu & Schnurbusch, 2012). There are many factors affecting wheat yield, among which tillering and spike differentiation are two key traits (Ao et al., 2010). The differentiation process of wheat young spike is a critical stage to achieve large spike size and more grains (Li et al., 2019). Spike development and growth have a great effect on the growth and survival of florets and the number of grains (Jiao et al., 2019; Waddington, Cartwright & Wall, 1983). Heading time is an important trait in the transition from vegetative to reproductive growth of cereal crops, which affects the adaptability of crops to various environmental conditions (Murai et al., 2003). Too high tillering level will impact the number of fertile spikes, and ultimately leads to a decrease in wheat yield (Kebrom et al., 2012). Spike development directly affects the number of fertile spikelets and grains per spikelet. Researches on wheat spikes have benefited from the large number of spike mutants. They can help us to understand the key genes and molecular mechanisms behind floret and grain developments (Sreenivasulu & Schnurbusch, 2012).

MiRNA is a type of single stranded non coding RNA with a length of about 20–25 nt. MiRNA widely exists in animals, plants and some viruses and plays roles in RNA silencing and post transcriptional regulation of gene expression (Zhu et al., 2022). The first microRNA, miRNA-*lin-4*, was discovered in the 1990s (Lee, Feinbaum & Ambros, 1993). Since then, microRNAs have been studied in many species including crops. A wheat small RNA library was constructed and identified 58 microRNAs (miRNAs). Northern blot analysis indicated that several of the newly identified miRNAs were preferentially expressed in specific tissues of wheat. Additionally, a significant number of monocot-specific miRNAs were also identified. These findings suggest that both conserved miRNAs and those unique to wheat play crucial roles in physiological processes, including wheat growth, development, and stress response (Yao et al., 2007). Transcription factors participate in many important cellular processes, such as signal transduction, morphogenesis, and environmental stress responses, by influencing or controlling the expressions of some specific genes (Chen & Cao, 2014). Some transcription factor families are unique in plants, such as WRKY (Eulgem et al., 2000), R2R3-MYB (Stracke, Werber & Weisshaar, 2001), NAC (Olsen et al., 2005), TIFY (Vanholme et al., 2007), SBP-box (Yang et al., 2008) *etc*.

Growth-regulating factors (GRFs) are a kind of plant-specific transcription factors. GRFs play multiple important regulatory roles in the growth and development of plants. They are identified for their roles in stem and leaf development, flower and seed formation, root development, and the coordination of growth processes under adverse environmental conditions (Bao et al., 2014; Debernardi et al., 2014; Hewezi et al., 2012; Liang et al., 2013; van der Knaap, Kim & Kende, 2000; Wu et al., 2014). The expressions of several *GRFs* are controlled by *miR396*, and the *miR396-GRFs* regulatory module appears to be central in several of these processes (Baucher et al., 2012; Liu et al., 2014; Omidbakhshfard et al., 2015; Rodriguez et al., 2015, 2010; Wang et al., 2011). Obviously, *miR396* and *GRFs* are two key gene expression regulation factors in plant species, however, wheat related studies are limited.

Previously, we found that the *miR396*-*TaGRFs* regulatory module played important roles in regulating wheat tillering (An et al., 2019; He et al., 2018; Zhang et al., 2021). However, their roles in wheat spike development are largely unknown. We obtained several spike mutants, including dwarf round spike mutant (*drs*), apical spikelet sterility mutant (*ass*) and prematurely terminated spike differentiation mutant (*ptsd1*) from the EMS-treated cultivar Guomai 301 (wild type, WT) (Jiao et al., 2019; Ni et al., 2015). In this study, we want to explore the roles of *tae-miR396b-TaGRFs* played in the abnormal wheat spike developments by analyzing the differential expressions of *tae-miR396b* and *TaGRFs* in WT and its mutants. Guomai 301 and its three mutants have the same genetic background, which is one advantage for study the roles of *miR396*-*TaGRFs* regulatory module as well as the effects of the mutated genes *drs*, *ass* and *ptsd1*. The results were reported here.

## Materials and Methods

### Plant materials and growth methods

Guomai 301 (WT) was bred in our laboratory, Henan Technology Innovation Centre of Wheat, Henan Agricultural University. The seeds of WT were treated with EMS and planted at Shuangba Experimental Station of Shangqiu Academy of Agricultural and Forestry Sciences (34°25′N, 115°39′E, 49 m above sea level). Where belongs to the continental monsoon climate zone. The seeds were treated with 0.4% EMS solution for 4 h, and then washed with tap water over night (Ni et al., 2015). The mutants *drs*, *ass* and *ptsd1* with spike—type variation were obtained in the M_2_ generation in 2014. Since 2014, the mutants have been planted and observed continuously in Xingyang experimental field in Zhengzhou (34°86′N, 113°44′E, 114 m above sea level) and Shuangba experimental station in Shangqiu. The mutants were at M11 generation in 2023. The field experiments were carried out in a completely randomized design as described by us (Duan et al., 2015).

### Identification and analysis of *tae-miR396b*

The *miR396b* sequences of wheat and other species were obtained by comparing and analyzing the *miR396b* sequences in miRBase (http://www.mirbase.org/) database. The *tae-miR396b* and their annotations were obtained by alignment analysis *miR396b* sequences in wheat EnsemblPlants (http://plants.ensembl.org/index.html) in wheat genome data. RNAfold software (http://unafold.rna.albany.edu/?q=mfold/RNA-Folding-Form) was used to predict the hairpin structures of *tae-miR396b* precursors. Sequence alignment analysis was carried out using DNAMAN 6.0 software (https://dnaman.software.informer.com/).

### Prediction of the *tae-miR396b* target sites on *TaGRFs*

The sequence of *tae-miR396b* was downloaded from the miRNA database (http://www.mirbase.org/). The *tae-miR396b* binding sites of *TaGRFs* were predicted on psRNATarget website (https://www.zhaolab.org/psRNATarget/). Parameters were chosen as the system default values.

### Tissue specific expression analysis of *TaGRFs*

The raw data of *TaGRFs’* expressions in various tissues and organs during wheat growth and development were downloaded from the Wheat Expression Browser website (http://www.wheat-expression.com/). Six samples of roots, leaves and spikes of Chinese Spring were analyzed at the 2nd node detectable (GS32) and boots just visibly swollen (GS43). The definition of wheat developmental stages were referred to the description of Zadoks, Chang & Konzak (1974). Tissue specific expression heatmaps of *TaGRFs* were generated using TBtools software. Expression profiles were drawn based on transcripts per million (TPM) values of *TaGRFs*.

### Real-time qRT-PCR

qRT-PCR was performed as described previously by us (An et al., 2019). The primers of *TaGRFs* were designed using Primer-Blast of NCBI website (https://www.ncbi.nlm.nih.gov/tools/primer-blast/). All the primer sequences were listed in Table S1. The *actin* gene was used as the internal control for real-time quantification of *TaGRFs*, and *U6* was used as the internal control for real-time quantification of *tae-miR396b*. Each reaction was performed with three technical repeats. The relative expressions of *TaGRFs* and *tae-miR396b* were calculated by 2^−ΔΔCt^ methods (Livak & Schmittgen, 2001).

### Correlation analysis between *tae-miR396b* and *TaGRFs* expressions in WT and mutants

Based on the results of qRT-PCR, Origin (https://www.originlab.com/) software was used to analyze the Pearson’s correlations of the expression levels of *tae-miR396b* and *TaGRFs* in WT and the mutants *ass*, *drs*, and *ptsd1*. The developmental stages were defined referred to that described by Vahamidis et al. (2014). Samples at three stages of the floret primordium visible stage, the two awns/spikelet reaching apical meristem of the spikelet stage, and the green anther stage (Vahamidis et al., 2014) were used to study the regulatory effect of *tae-miR396b* on different *TaGRFs*.

### Expression vector construction and interaction analysis of *tae-miR396b* and *TaGRF2-7A*

According to the precursor sequence of *tae-miR396b*, the primers with *Spe*I restriction site were designed for cloning *tae-miR396b* and construction the expression vector pCAMBIA1304-*tae-miR396b* (Table S1). According to the CDS of *TaGRF2-7A*, the primers with *Spe*I restriction site were designed for cloning the full length cDNA of *TaGRF2-7A* and construction the expression vector pCAMBIA1304-*TaGRF2-7A* (Table S1). The *pre-miR396b* and CDS of *TaGRF2-7A* sequences were amplified using the genomic DNA and cDNA of Guomai 301 respectively. The *pre-miR396b* and CDS of *TaGRF2-7A* were constructed into the expression vector pCAMBIA1304 and formed pCAMBIA1304-*35S::pre-miR396b* and pCAMBIA1304-*35S::TaGRF2-7A*. The pCAMBIA1304-*35S::pre-miR396b* and pCAMBIA1304-*35S::TaGRF2-7A* were transformed into GV3101 strain (*Agrobacterium tumefaciens*). 5-week-old tobacco (*Nicotiana benthamiana*) leaves were infiltrated with pCAMBIA1304-*35S::TaGRF2-7A* (P1) or pCAMBIA1304 (P3) alone, and co-transformed with pCAMBIA1304-*35S::pre-miR396b* and pCAMBIA1304-*35S::TaGRF2-7A* (P2). Tobacco leaves with different treatments at 2, 24, 48, and 72 h post-infiltration were sampled respectively. The dynamic changes of *TaGRF2-7A* expression were analyzed by qRT-PCR. *Actin* gene was used as an internal control.

### GUS staining

Tobacco was transformed with *Agrobacterium* carrying plasmid pCAMBIA1304-*35S::TaGRF2-7A*, and co-transformed with *Agrobacterium* carrying plasmid pCAMBIA1304-*35S::TaGRF2-7A* and *Agrobacterium* carrying plasmid pCAMBIA1304-*35S::pre-miR396b* separately. The tobacco leaves at 48 h after transformation with the expression vectors were sampled and stained with GUS staining buffer at 37 °C overnight. The stained tobacco leaves were then placed in 70% alcohol in a 60 °C water bath for 10 min, and then the tobacco leaves were placed in anhydrous ethanol in a 60 °C water bath until the tobacco leaves were completely decolorized (Yang, Poretska & Sieberer, 2018).

## Results

### Spike phenotypes of WT, *ptsd1*, *drs*, and *ass*

**Figure 1:**
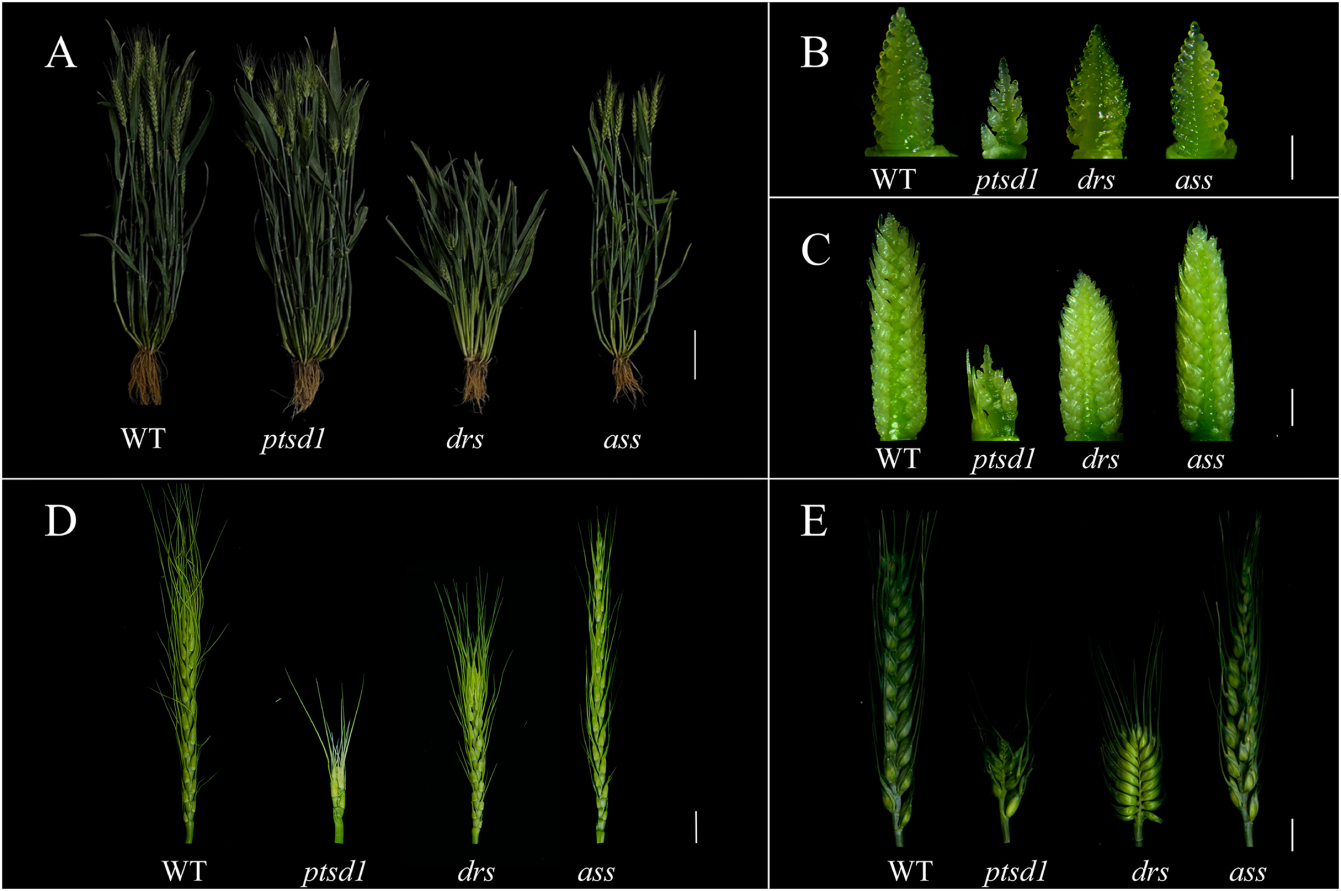
Comparison of plant and spike morphology between WT and mutant *ptsd1*, *drs*, and *ass* (left—right). (A) The plants of WT, *ptsd1*, *drs*, and *ass* at the grain filling stage (left—right). (B) The spikelets of WT, *ptsd1*, *drs*, and *ass* at the floret primordium visible stage. (C) The spikelets of WT, *ptsd1*, *drs*, and *ass* at the two awns/spikelet reaching apical meristem of the spikelet stage. (D) The spikelets of WT, *ptsd1*, *drs*, and *ass* at the green anther stage; (E) The spikes of WT, *ptsd1*, *drs*, and *ass* at grain filling stage. (A) Scale bar = 10 cm; (B) and (C) Scale bar = 1 mm; (D) and (E) Scale bar = 1 cm. The developmental stages were defined referred to that described by Vahamidis et al. (2014).

**Figure 2:**
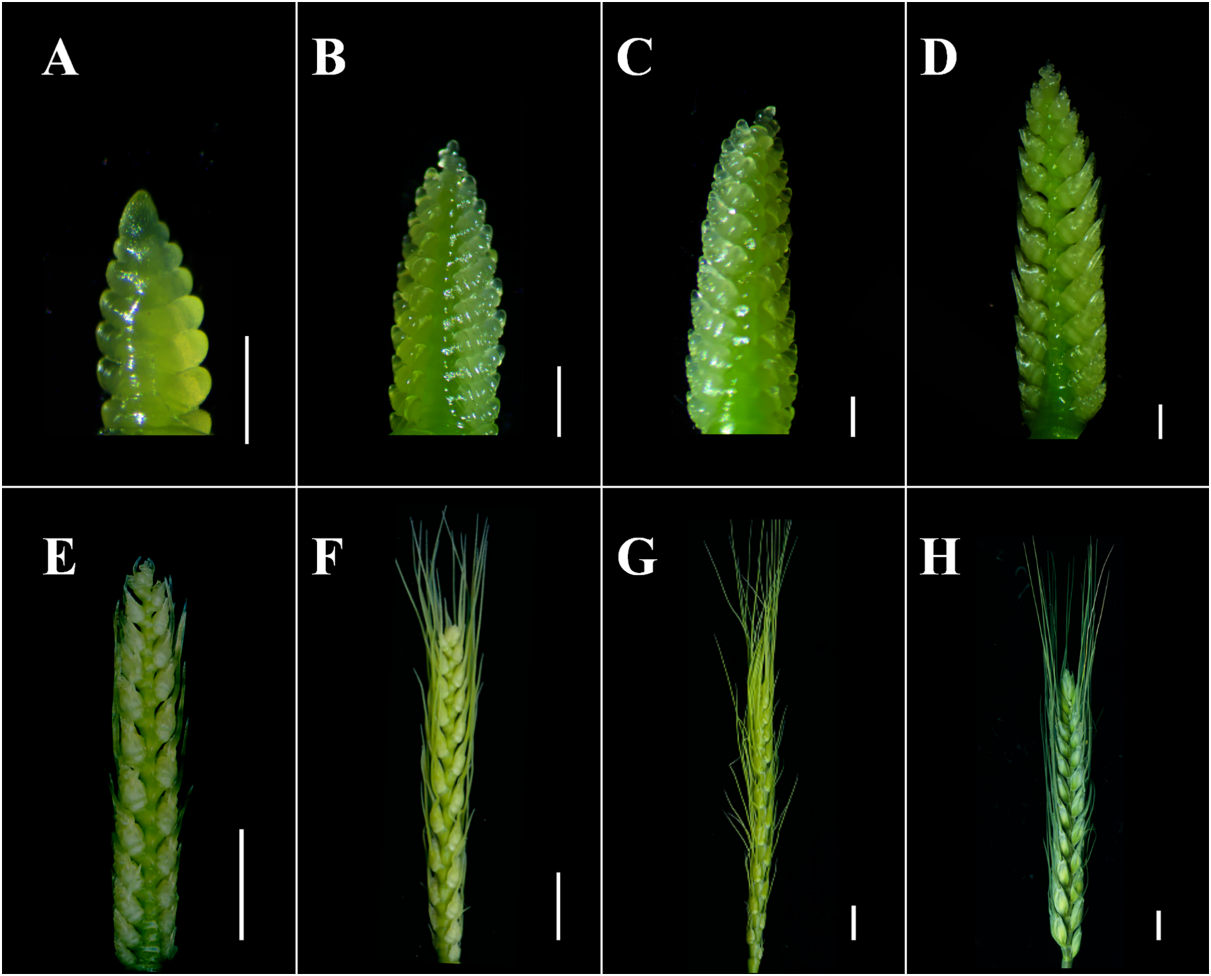
Illustration of the proposed scales of wheat spike differentiation of WT. (A) Glume primordium visible. (B) Floret primordium visible stage. (C) Terminal spikelet stage. (D) Two awns/spikelet reaching apical meristem of the spikelet stage. (E) Two basal florets fully covered by lemmas stage. (F) Spikelet apical meristem just visible or white anther stage. (G) Green anther stage. (H) Grain filling stage. (A–D) Scale bar = 1 mm. (E–H) Scale bar = 1 cm. The developmental stages were defined referred to that described by Vahamidis et al. (2014).

The agronomic traits between WT and the mutants *ptsd1*, *drs*, and *ass* were compared. There was no significant difference in plant height between WT and *ptsd1* at grain filling stage. However, there were significant differences in spike component traits (Jiao et al., 2019). Compared to WT, the spike length was significantly shorter and spikelets per spike were fewer in *ptsd1* (Figs. 1A and 1E; Table 1). The upper spikelets of *ptsd1* stopped differentiating, but the basal spikelets could still differentiate at the floret primordium visible stage and the two awns/spikelet reaching apical meristem of the spikelet stage (Figs. 1B and 1C). At the green anther stage, the upper spikelets could not form stamens due to the stopped spikelet differentiation, resulting in a significantly fewer fertile spikelets than that of WT (Fig. 1D). At the grain filling stage, the plant height and spike length were significantly shorter in mutant *drs* than that in WT, but the spikes of *drs* were wider (Figs. 1A and 1E). At the floret primordium visible stage, the bottom of the spike was significantly wider than the top of the spike (Fig. 1B). There was already a clear difference in spike length between WT and *drs* at the two awns/spikelet reaching apical meristem of the spikelet stage (Fig. 1C). At the green anther stage, the spike length was significantly shorter than that of WT, and finally the whole spike was oval (Fig. 1D). At the grain filling stage, the mutant *ass* was slightly shorter than that of WT in plant height, and the fertile spikelets were significantly fewer than that of WT due to the pistil and stamen degenerations of the apical spikelets (Figs. 1A and 1E; Table 1). At the floret primordium visible stage and the two awns/spikelet reaching apical meristem of the spikelet stage, the apical spikelets of *ass* developed normally, and there was no significant difference between *ass* and WT (Figs. 1B and 1C). However, at the green anther stage, the apical spikelets of *ass* have developed abnormally, and finally the apical spikelets could not developed and set grain normally (Fig. 1D). Wheat spike developmental stages were described as following (Fig. 2; Vahamidis et al., 2014).

**Table 1:** Comparison of agronomic traits between the WT and mutants *ptsd1*, *drs* and *ass*.

Tract	WT	*drs*	*ass*	*ptsd1*
Plant height/cm	64.45 ± 3.28	38.52 ± 3.15**	53.65 ± 3.34*	50.75 ± 3.09**
Tiller number	20.73 ± 3.20	23.82 ± 2.20	16.77 ± 2.32	21.73 ± 2.30
Spike length/cm	11.47 ± 0.53	4.25 ± 0.88**	12.13 ± 0.67	3.86 ± 1.30**
Spike width/cm	2.11 ± 0.13	2.39 ± 0.22**	1.76 ± 0.21*	1.48 ± 0.10**
Spikelet number	21.00 ± 2.00	17 ± 2.00*	21.00 ± 2.00	4.50 ± 1.50**
Fertile spikelet number	20.00 ± 1.00	17 ± 2.00*	12 ± 2.00**	4.50 ± 1.50**

**Notes:**

Phenotypic performance of each trait was expressed as means ± standard deviation (*n* = 10).

** and *, significant difference at *P* < 0.01 and *P* < 0.05 by *t*-test, respectively.

### *Tae-miR396b* was located on the long arms of chromosomes 6A, 6B and 6D

**Figure 3:**
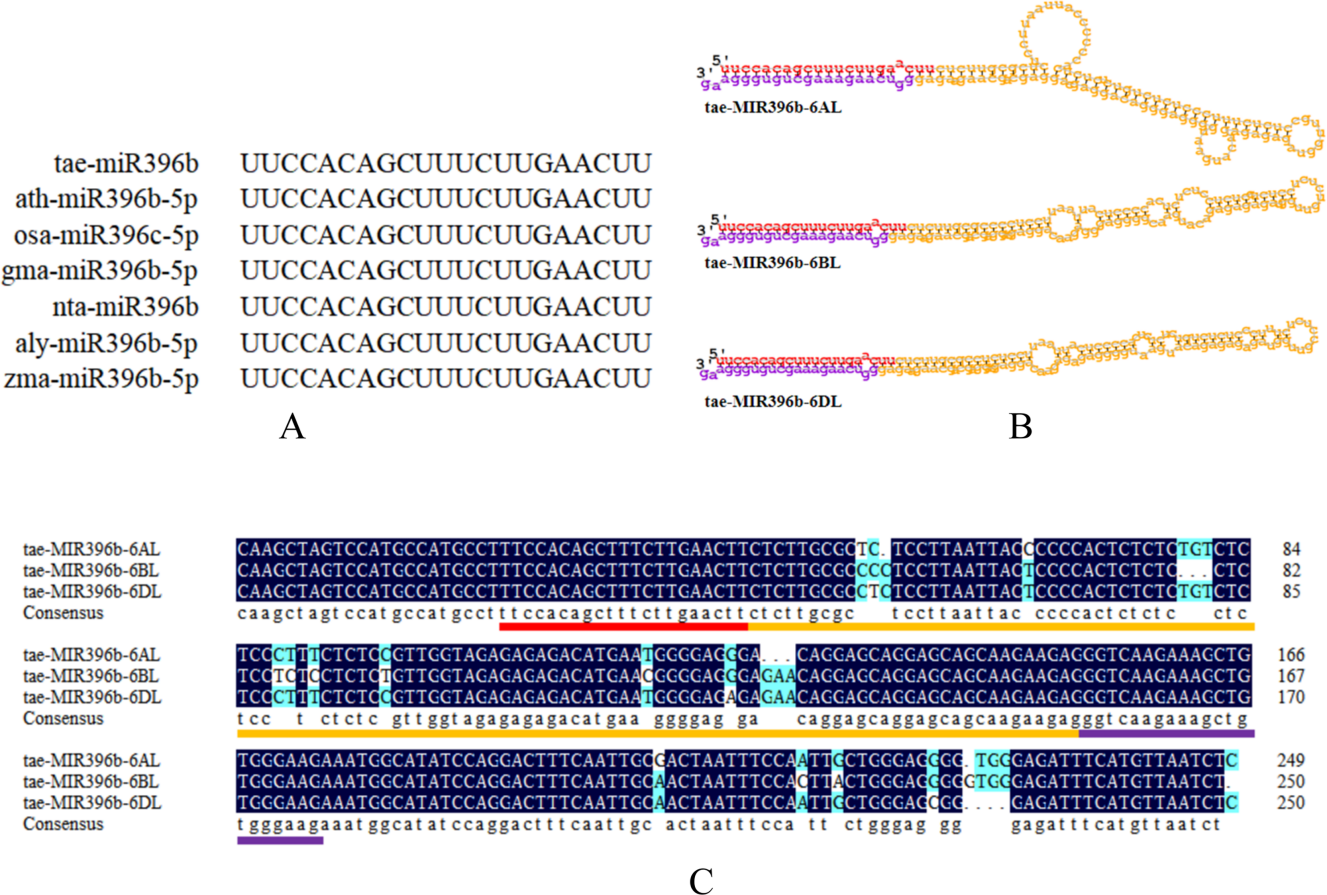
Characteristic information of *tae-miR396b*. (A) Sequence alignment of the mature *miR396s* in wheat (*T. aestivum* L.; tae-), Arabidopsis (*A*. *thaliana*; ath-), rice (*O. sativa*; osa-), soybean (*G. max*; gma-), tobacco (*N. tabacum*; nta-), *Arabidopsis lyrata* (*A. lyrata*; aly-) and maize (*Z. mays*; zma-). (B) Stem-loop structures of *tae-MIR396b-6AL*, *tae-MIR396b-6BL* and *tae-MIR396b-6DL*. The red part is mature sequence. The yellow part is the stem-loop sequence. The purple part is star sequence. (C) Sequence alignment of the three *tae-MIR396b* genes. The places marked with light blue are different sequences (SNPs and InDels).

To explore the evolution of *tae-miR396b*, the sequence alignment of *tae-miR396b* with the homologs in other species were carried out. The result indicated that the *miR396bs* of wheat (*T. aestivum* L.), Arabidopsis (*Arabidopsis thaliana*), rice (*Oryza sativa*), soybean (*Glycine max*), tobacco (*Nicotiana tabacum*), *Arabidopsis lyrata*, and maize (*Zea mays*) were highly conserved (Fig. 3A). Mature miRNA is incorporated into the miRNA-induced silencing complex (miRISC). As part of miRISC, miRNAs base-pairs target miRNAs and induce their translational repression or deadenylation and degradation. Sequence exploration of miR396b in wheat genome indicated that there were three homeologous genes of *tae-miR396b* on the long arms of chromosomes 6A, 6B and 6D (Fig. 3B), and they were named as *tae-miR396b-6AL*, *tae-miR396b-6BL*, and *tae-miR396b-6DL* respectively. The sequences of the three homeologous genes were highly conserved except for some SNPs and InDels among them. However, they encoded the same mature *tae-miR396b* (Fig. 3C).

### Twenty-three *TaGRFs* had binding sites of *tae-miR396b*

**Figure 4:**
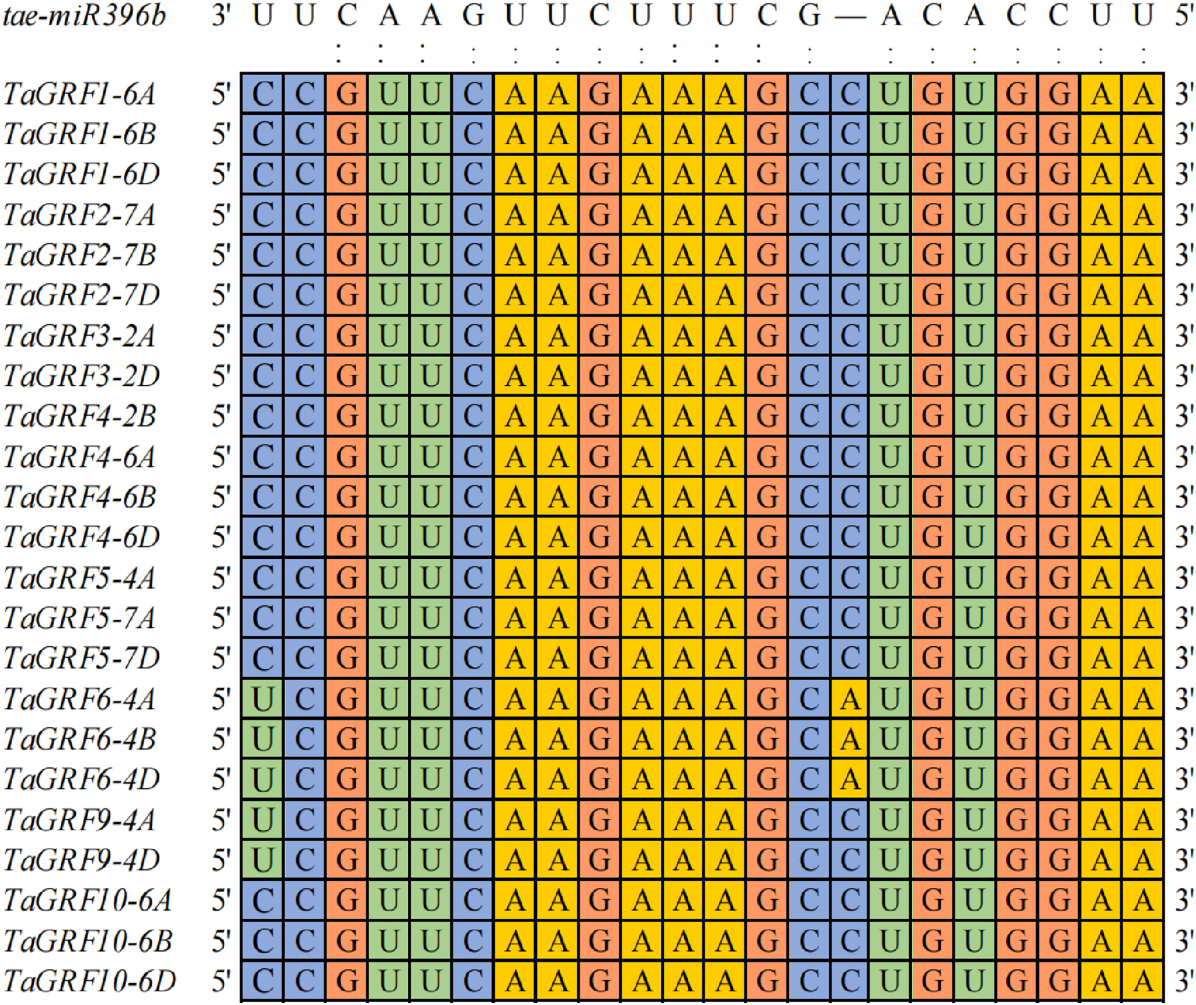
Sequence alignment of *tae-miR396b* with complementary sequences in *TaGRF* genes.

To explore how many genes were the targets of *tae-miR396b*, the binding sites of *tae-miR396b* were screened in all wheat genes. A total of 203 target genes of *tae-miR396b* were identified in wheat genome (Table S2). Among the target genes, 23 genes belonged to the *GRF* gene family. Wheat *GRF* gene family has 30 genes (Zhang et al., 2021). The sequence alignment of the 30 *TaGRFs* with *tae-miR396b* (*tae-miR396b*: 5′-UUCCACAGCUUUCUUGAACUU-3′) further confirmed that the 23 *TaGRFs* had the binding sites of *tae-miR396b* (Fig. 4). This result demonstrated that the most *TaGRF* genes were the potential targets of *tae-miR396b*.

### *TaGRFs* were highly expressed in spikes

**Figure 5:**
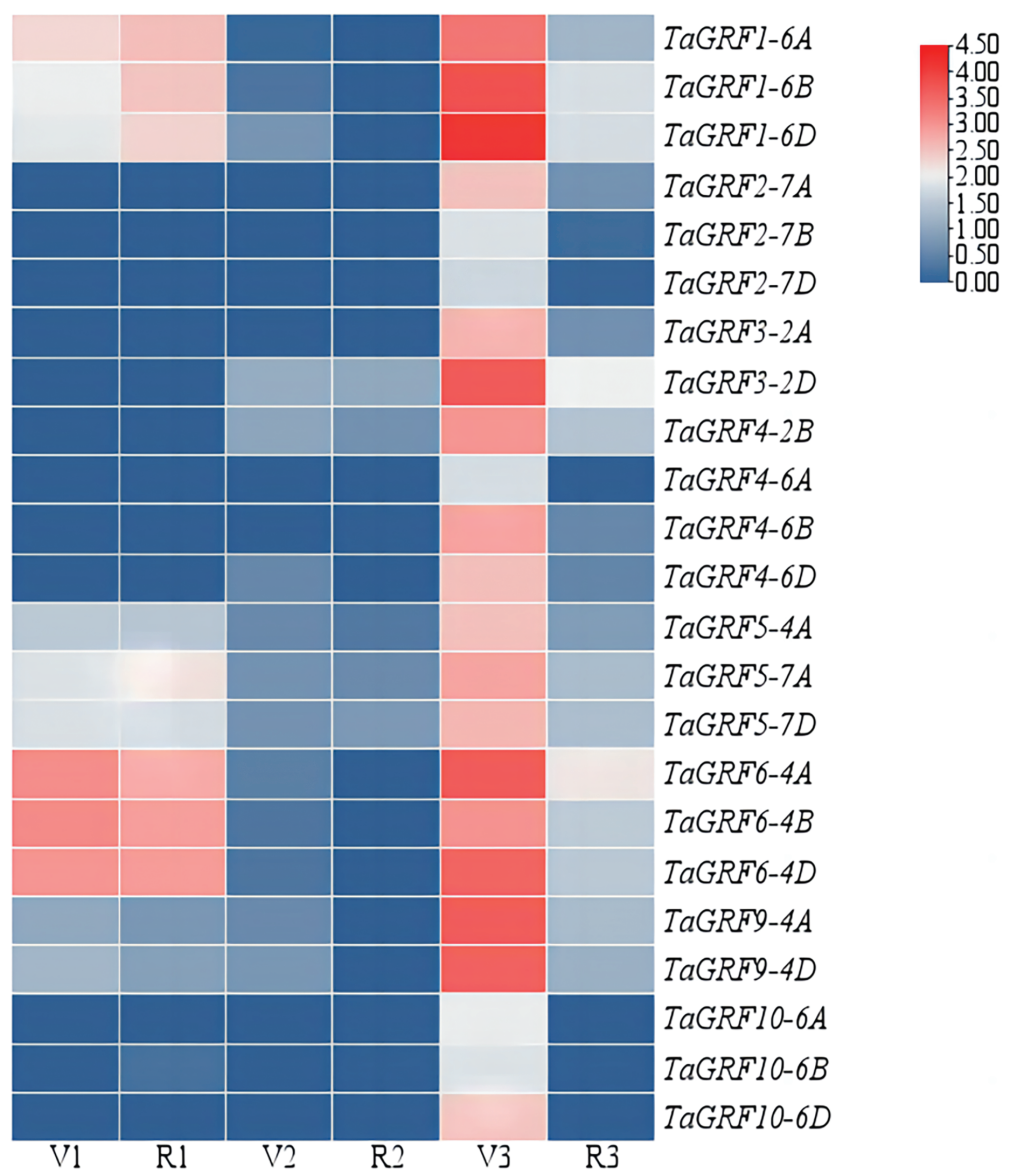
Expression levels of the *TaGRF* genes at 2nd node detectable (GS32) and boots just visibly swollen (GS43). V1, Roots of Chinese Spring at GS32; V2, leaves of Chinese Spring at GS32; V3, spikes of Chinese Spring at GS32; R1, roots of Chinese Spring at GS43; R2, leaves of Chinese Spring at GS43; R3, spikes of Chinese Spring at GS43.

The expressions of the *TaGRFs* in roots, leaves and spikes of Chinese Spring at GS32 and GS43 stage were analyzed (Fig. 5 and Table S3). The results showed that *TaGRF1* (*6A*, *6B*, *6D*), *TaGRF5* (*4A*, *7A*, *7D*) and *TaGRF6* (*4A*, *4B*, *4D*) were consistently expressed at high levels during root development. *TaGRF2* (*7A*, *7B, 7D*), *TaGRF3* (*2A*, *2D*), *TaGRF4* (*2B*, *4A*, *4B*, *4D*) and *TaGRF10* (*6A*, *6B*, *6D*) were lowly expressed or even couldn’t be detected during root development. The expression levels of the 23 *TaGRFs* were low or even not expressed in leaves. However, during spike development, all the *TaGRFs* expressed at a high level at GS32, but the expression levels decreased or some *TaGRFs* were even not expressed at GS43. The above results indicated that *TaGRFs* were highly expressed in the process of spike development, and the expression levels varied greatly in different developmental stages. Because wheat spikes were at early differentiation stages at GS32, completed differentiation at GS43, *TaGRFs* played more important roles in spike differentiation.

### Functional classification of the *TaGRFs* in GO

Gene Ontology annotation analysis of *TaGRFs* showed that all *TaGRFs* played the same functions in molecular functions, biological processes and cellular components (Table 2). They were mainly involved in ATP binding (GO:0005524), regulation of DNA-templated transcription (GO:0006355), and developmental processes (GO:0032502).

**Table 2:** Functional classification of the *TaGRFs* in GO.

	Gene ID	+ no	Molecular function	Cellular component	Biological process		
TaGRF1-6A	TraesCS6A01G335900	5	GO:0005524	GO:0005634	GO:0006351	GO:0006355	GO:0032502
TaGRF1-6B	TraesCS6B01G366700	5	GO:0005524	GO:0005634	GO:0006351	GO:0006355	GO:0032502
TaGRF1-6D	TraesCS6D01G315700	5	GO:0005524	GO:0005634	GO:0006351	GO:0006355	GO:0032502
TaGRF2-7A	TraesCS7A01G165600	5	GO:0005524	GO:0005634	GO:0006351	GO:0006355	GO:0032502
TaGRF2-7B	TraesCS7B01G070200	5	GO:0005524	GO:0005634	GO:0006351	GO:0006355	GO:0032502
TaGRF2-7D	TraesCS7D01G166400	5	GO:0005524	GO:0005634	GO:0006351	GO:0006355	GO:0032502
TaGRF3-2A	TraesCS2A01G435100	5	GO:0005524	GO:0005634	GO:0006351	GO:0006355	GO:0032502
TaGRF3-2D	TraesCS2D01G435200	5	GO:0005524	GO:0005634	GO:0006351	GO:0006355	GO:0032502
TaGRF4-2B	TraesCS2B01G458400	5	GO:0005524	GO:0005634	GO:0006351	GO:0006355	GO:0032502
TaGRF4-6A	TraesCS6A01G269600	5	GO:0005524	GO:0005634	GO:0006351	GO:0006355	GO:0032502
TaGRF4-6B	TraesCS6B01G296900	5	GO:0005524	GO:0005634	GO:0006351	GO:0006355	GO:0032502
TaGRF4-6D	TraesCS6D01G245300	5	GO:0005524	GO:0005634	GO:0006351	GO:0006355	GO:0032502
TaGRF5-4A	TraesCS4A01G434900	5	GO:0005524	GO:0005634	GO:0006351	GO:0006355	GO:0032502
TaGRF5-7A	TraesCS7A01G049100	5	GO:0005524	GO:0005634	GO:0006351	GO:0006355	GO:0032502
TaGRF5-7D	TraesCS7D01G044200	5	GO:0005524	GO:0005634	GO:0006351	GO:0006355	GO:0032502
TaGRF6-4A	TraesCS4A01G255000	5	GO:0005524	GO:0005634	GO:0006351	GO:0006355	GO:0032502
TaGRF6-4B	TraesCS4B01G060000	5	GO:0005524	GO:0005634	GO:0006351	GO:0006355	GO:0032502
TaGRF6-4D	TraesCS4D01G059600	5	GO:0005524	GO:0005634	GO:0006351	GO:0006355	GO:0032502
TaGRF9-4A	TraesCS4A01G291500	5	GO:0005524	GO:0005634	GO:0006351	GO:0006355	GO:0032502
TaGRF9-4D	TraesCS4D01G020300	5	GO:0005524	GO:0005634	GO:0006351	GO:0006355	GO:0032502
TaGRF10-6A	TraesCS6A01G257600	5	GO:0005524	GO:0005634	GO:0006351	GO:0006355	GO:0032502
TaGRF10-6B	TraesCS6B01G267500	5	GO:0005524	GO:0005634	GO:0006351	GO:0006355	GO:0032502
TaGRF10-6D	TraesCS6D01G238900	5	GO:0005524	GO:0005634	GO:0006351	GO:0006355	GO:0032502

**Note:**

ATP binding (GO:0005524); nucleus (GO:0005634); DNA-templated transcription (GO:0006351); regulation of DNA-templated transcription (GO:0006355); developmental process (GO:0032502).

### Expression profiles of *tae*- *miR396b* and *TaGRFs* in WT, *drs*, *ass*, and *ptsd1*

**Figure 6:**
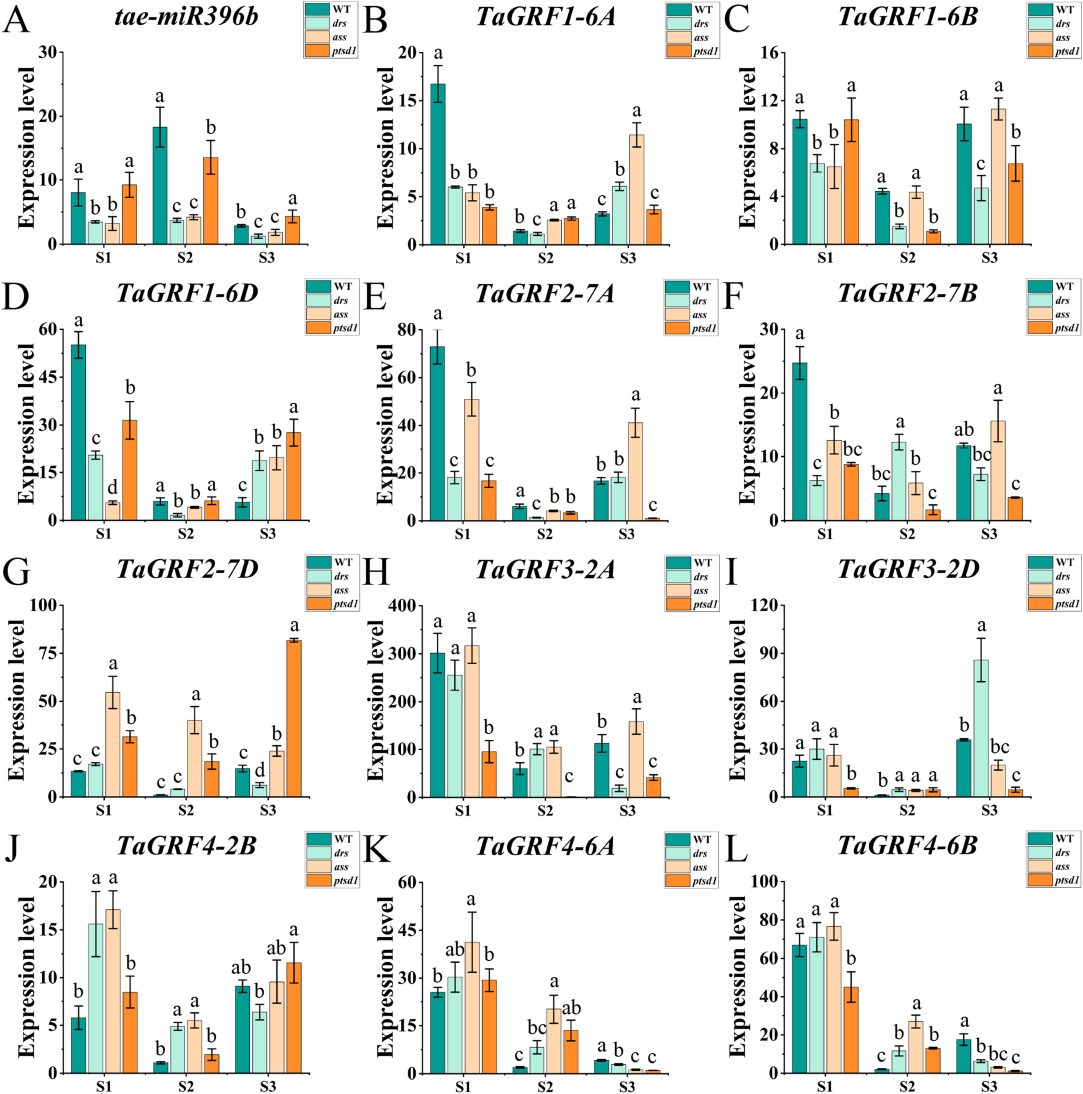
Expression analysis of *tae-miR396b* and 11 *TaGRF* genes at different stages by qRT-PCR. (A–L) The qRT-PCR results of *tae-miR396b* and 11 *TaGRF* genes in WT, *ptsd1*, *drs*, and *ass* at three different stages. S1, floret primordium visible stage; S2, two awns/spikelet reaching apical meristem of the spikelet stage; S3, green anther stage. The internal reference of *tae-miR396b* was *U6* gene, and the internal reference of 11 *TaGRF* genes were *actin* gene. Vertical bars indicated standard deviation. Bars (means) with different letters are significantly different (*P* < 0.01).

**Figure 7:**
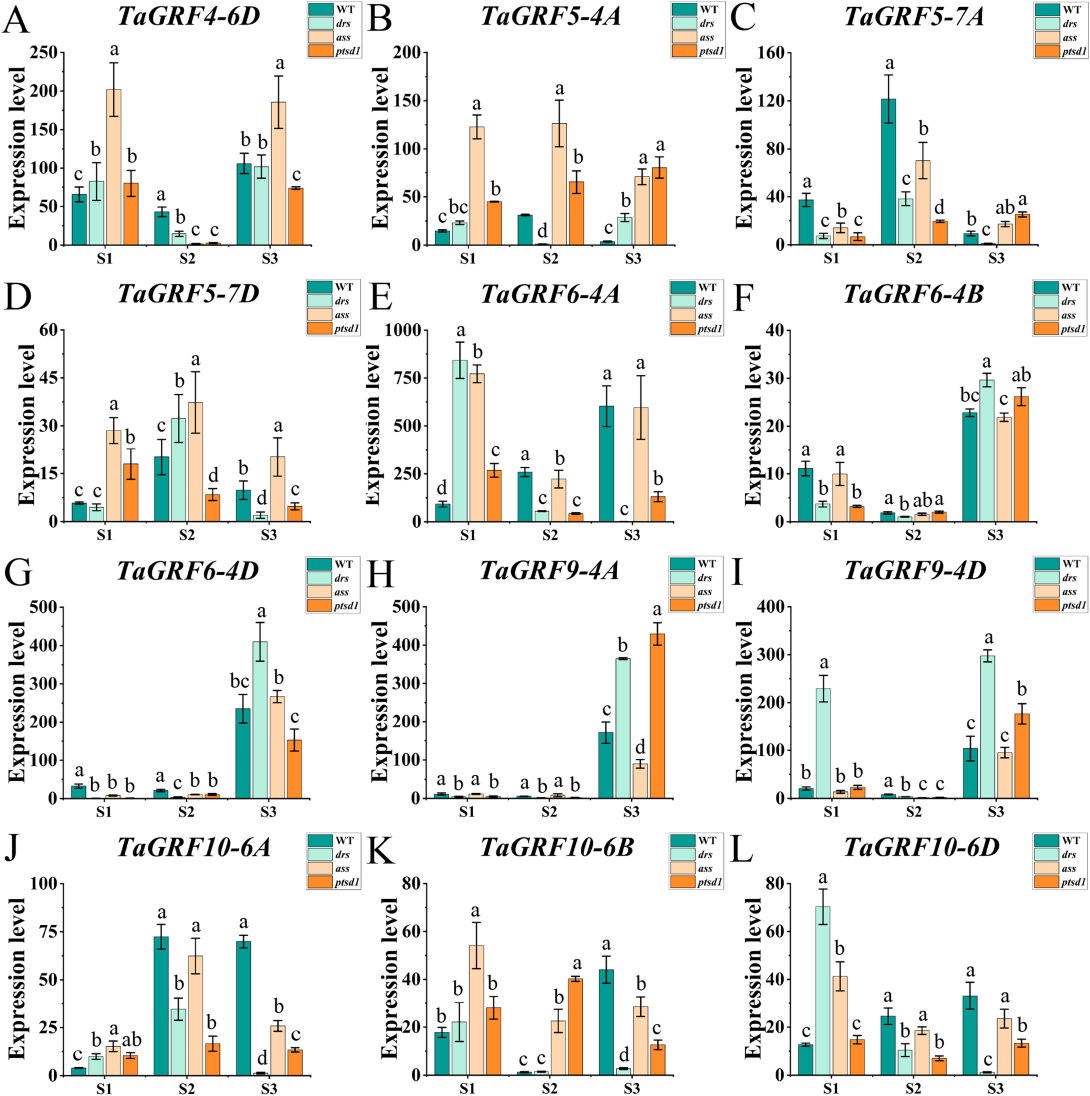
Expression analysis of 12 *TaGRF* genes at different stages by qRT-PCR. (A–L) The qRT-PCR results of 12 *TaGRFs* genes in WT, *ptsd1*, *drs*, and *ass* at three different stages. S1, floret primordium visible stage; S2, two awns/spikelet reaching apical meristem of the spikelet stage; S3, green anther stage. Data were normalized to *actin* gene, and vertical bars indicated standard deviation. Bars (means) with different letters are significantly different (*P* < 0.01).

The qRT-PCR was performed to analyze the expression profiles of *tae-miR396b* and *TaGRFs* in spikelets of WT and mutants *drs, ass*, and *ptsd1* at three spikelet developmental stages. Expression profiles of *tae-miR396b* and *TaGRFs* in the spikes of WT and mutants were different at the floret primordium visible stage, the two awns/spikelet reaching apical meristem of the spikelet stage, and the green anther stage. The relative expression levels of *tae-miR396b* and *TaGRFs* were also changed at different stages. Generally, the expression patterns of *tae*-*miR396b* and most *TaGRFs* were similar, but their expression levels were significantly different (Figs. 6 and 7).

The real-time quantitative analysis revealed that the expression of *tae-miR396b* initially increased and then decreased in WT and *ptsd1*, while little change in expression in *drs* and *ass*. Notably, the expression level of *tae-miR396b* was significantly higher in WT compared to mutants *drs* and *ass*. In WT, the expression levels of *tae-miR396b* were significantly different at the three stages. Most *TaGRFs* were highly expressed at S1, decreased at S2 and then increased again at S3, especially *TaGRF1* (*6A*, *6B*, *6D*), *TaGRF2* (*7A, 7B, 7D*), *TaGRF3* (*2A, 2D*), *TaGRF4-2B* and *TaGRF6-4A*. The expression levels of *TaGRF1* (*6A*, *6B*, *6D*), *TaGRF2* (*7A, 7D*), *TaGRF3-2D, TaGRF4-6D*, *TaGRF6* (*4A, 4B, 4D*) and *TaGRF9* (*4A*, *4D*) were all significantly increased at S3, implying they played roles in regulating spike development. The expression levels of *TaGRF1* (*6A, 6D*), *TaGRF2* (*7A, 7B, 7D*), *TaGRF4* (*6A, 6B*), *TaGRF5* (*4A, 7A, 7D*), and *TaGRF10-6* (*A. B, D*) between WT and the mutants were significantly different at S1, S2 and S3, implying they might be the causes of abnormal spike differentiation in the mutants. In summary, the expression levels of most *TaGRFs* were opposite to that of *tae-miR396b*, which demonstrated that the expressions of most *TaGRFs* were negatively regulated by *tae-miR396b*.

### *Tae-miR396b* had different regulatory effects on *TaGRFs* in WT and mutants *drs*, *ass* and *ptsd1*

**Figure 8:**
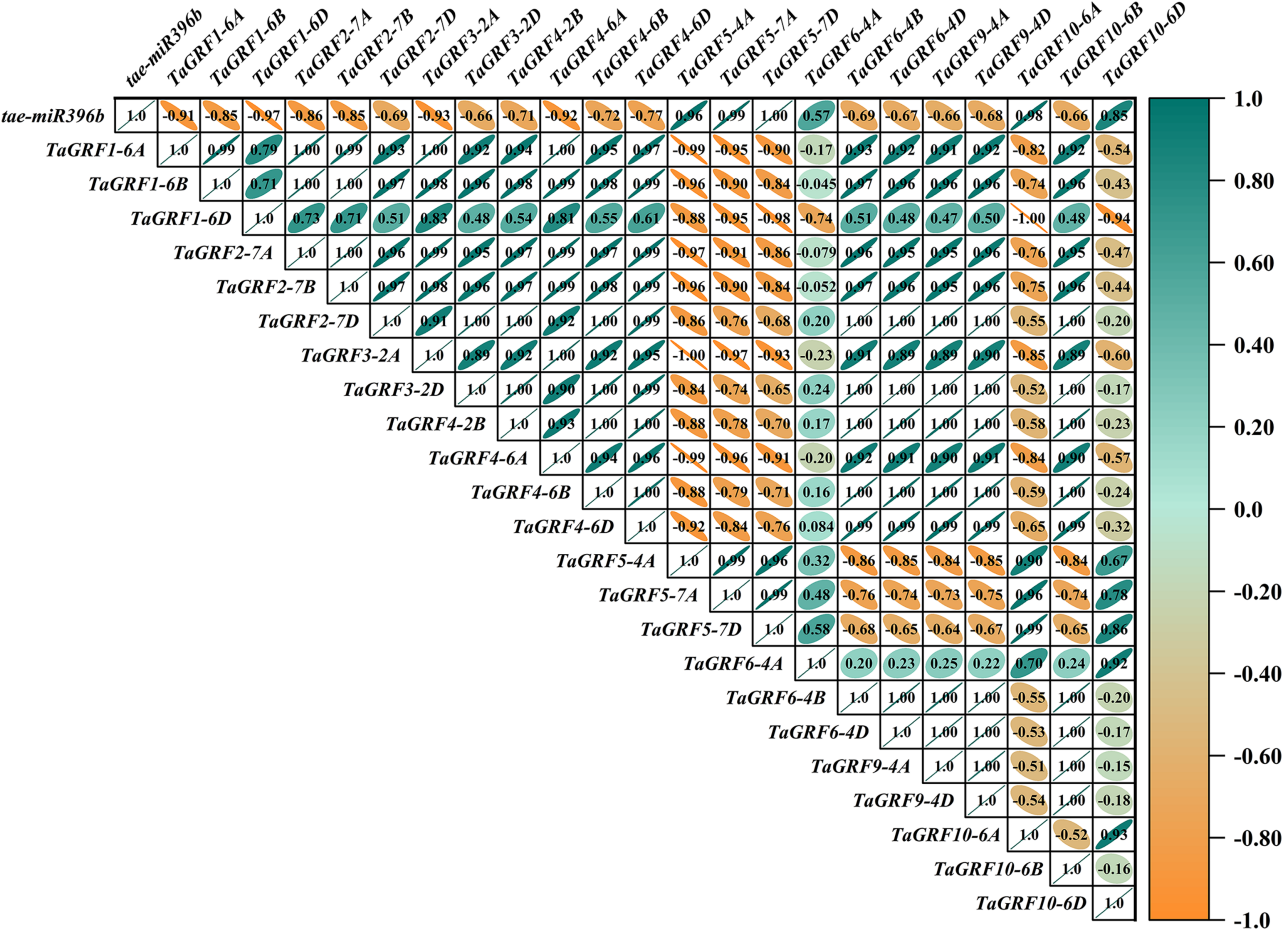
Heat map of the correlations between *tae-miR396b* and *TaGRFs* expressions in WT. The color scale indicates the values of Pearson’s rank correlation coefficient. Orange indicates a negative correlation; green indicates a positive correlation. The numbers in the figure indicate the correlation coefficients.

**Figure 9:**
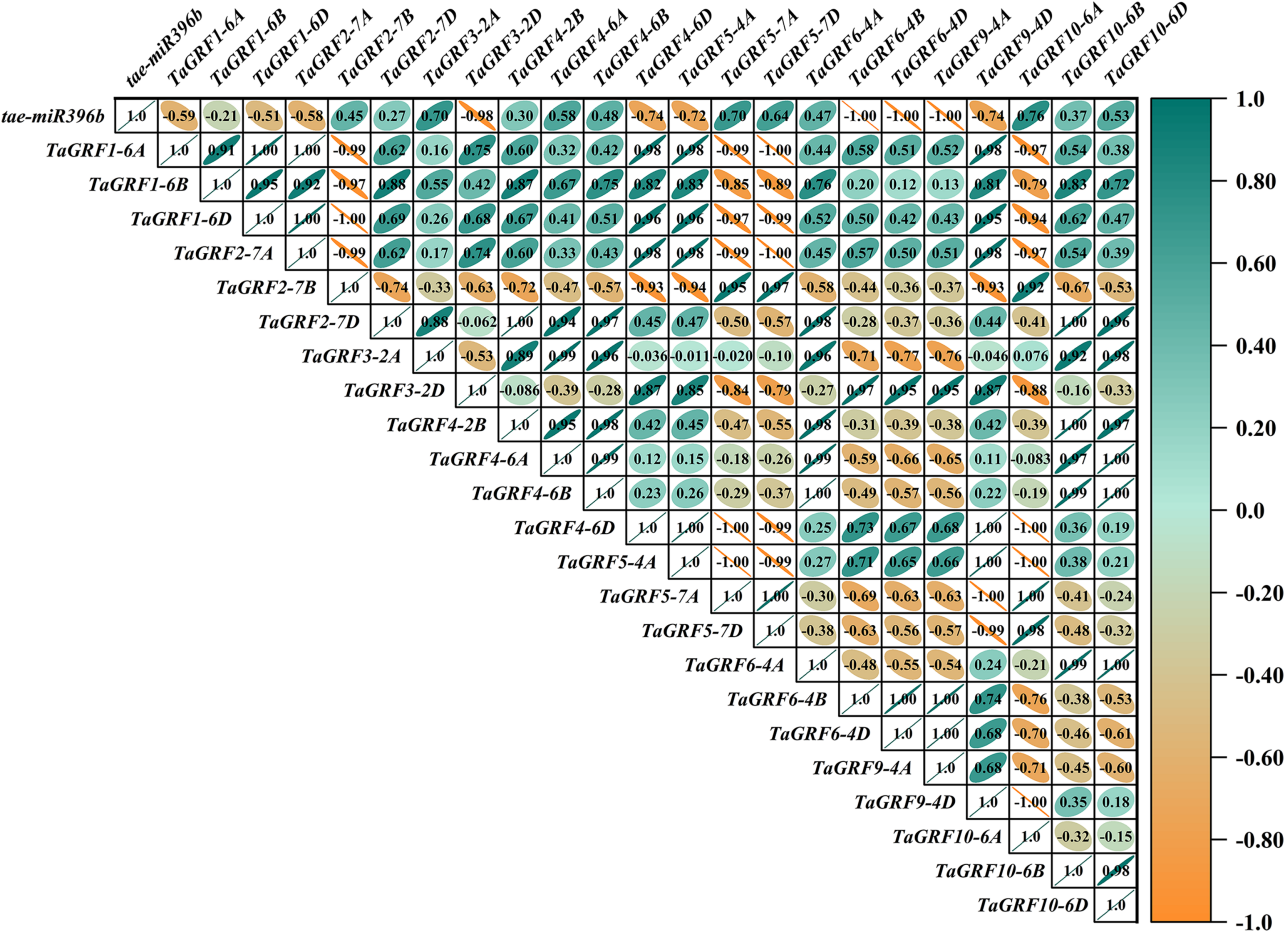
Heat map of the correlations between *tae-miR396b* and *TaGRFs* expressions in *drs*. The color scale indicates the values of Pearson’s rank correlation coefficient. Orange indicates a negative correlation; green indicates a positive correlation. The numbers in the figure indicate the correlation coefficients.

**Figure 10:**
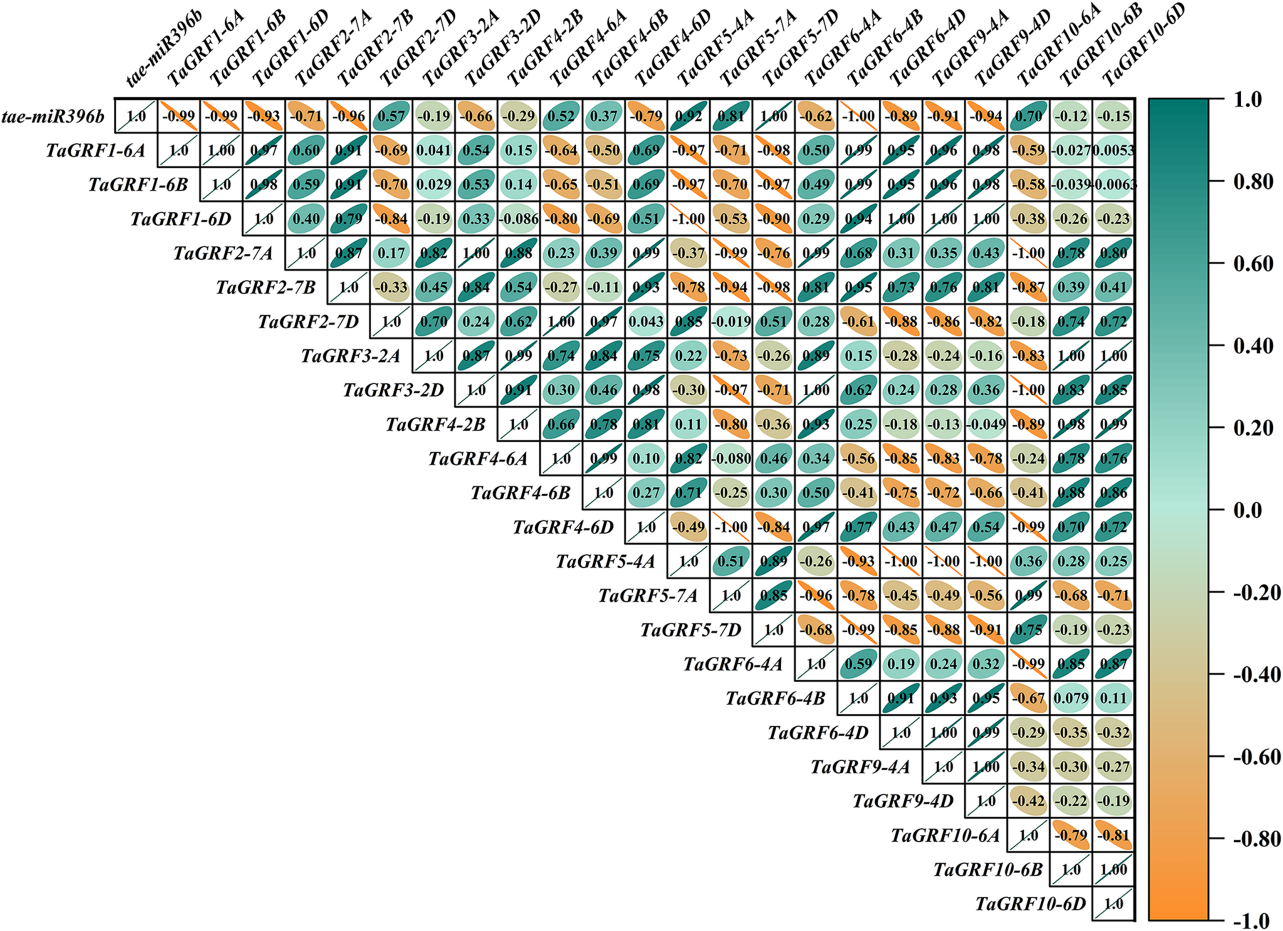
Heat map of the correlations between *tae-miR396b* and *TaGRFs* expressions in *ass*. The color scale indicates the values of Pearson’s rank correlation coefficient. Orange indicates a negative correlation; green indicates a positive correlation. The numbers in the figure indicate the correlation coefficients.

**Figure 11:**
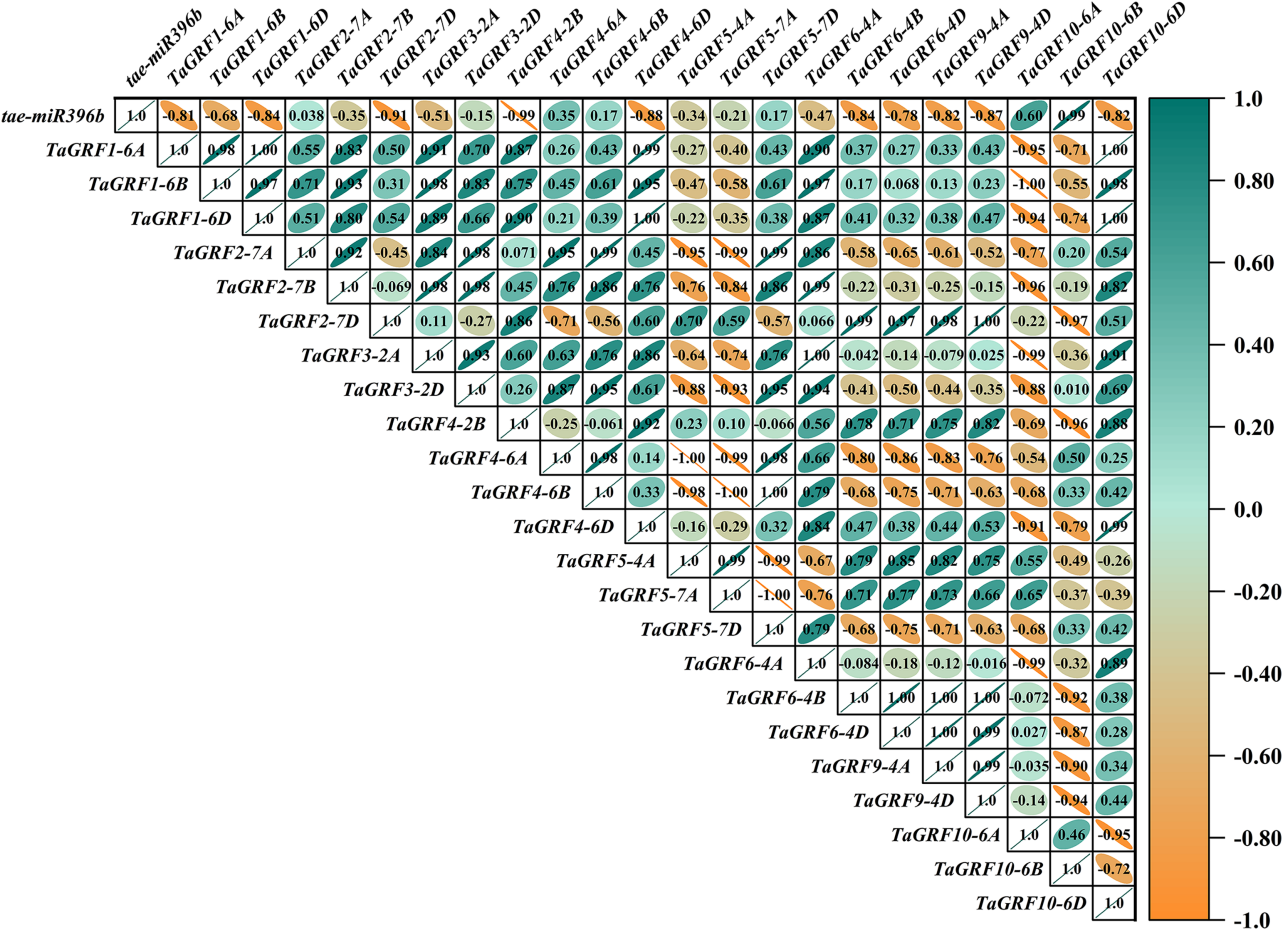
Heat map of the correlations between *tae-miR396b* and *TaGRFs* expressions in *ptsd1*. The color scale indicates the values of Pearson’s rank correlation coefficient. Orange indicates a negative correlation; green indicates a positive correlation. The numbers in the figure indicate the correlation coefficients.

In WT, the expression of *tae-miR396b* was negatively correlated with the expressions of *TaGRF1* (*6A*, *6B*, *6D*), *TaGRF2* (*7A, 7B, 7D*) and *TaGRF3* (*2A, 2D*) (Fig. 8). In *drs*, the expression of *tae-miR396b* was significantly negatively correlated with the expressions of 7 *TaGRFs* including *TaGRF3-4D*, *TaGRF4-6D, TaGRF5-4A, TaGRF4* (*6B*, *6D*), *TaGRF9-4A* and *TaGRF10-6A* (Fig. 9). In *ass*, the expression of *tae-miR396b* was negatively correlated with the expressions of most *TaGRFs* including *TaGRF1* (*6A*, *6B*, *6D*), *TaGRF2* (*7A, 7B*), *TaGRF3*-*2D*, *TaGRF4-6D, TaGRF6* (*4B, 4D*) and *TaGRF9* (*4A, 4D*) (Fig. 10). In *ptsd1*, the expression of *tae-miR396b* was negatively correlated with the expressions of *TaGRF1* (*6A*, *6B*, *6D*), *TaGRF2-7D*, *TaGRF4* (*2B, 6D*), *TaGRF6* (*4D, 4D*), *TaGRF9* (*4A*, *4D*) and *TaGRF10-6D* (Fig. 11). These results demonstrated that *tae-miR396b* had different regulatory effects on *TaGRFs* in WT and the mutants. The expression patterns of *TaGRF* genes were complex. Both *tae-miR396b* and *TaGRF* genes impact mutant spike development to some extent.

Correlation analysis showed that *tae-miR396b* was negatively correlated with most *TaGRFs*, however, it was positively correlated with some *TaGRFs*, especially in mutant *drs*. The reason might be that the expressions of *TaGRFs* were not only regulated by *tae-miR396b*, but also regulated by other factors, because there were many *cis*-elements in the promoters of *TaGRFs*. Obviously, the expression regulations of *TaGRF* genes were more complex.

### *TaGRF2-7A* was negatively regulated by *tae-miR396b*

**Figure 12:**
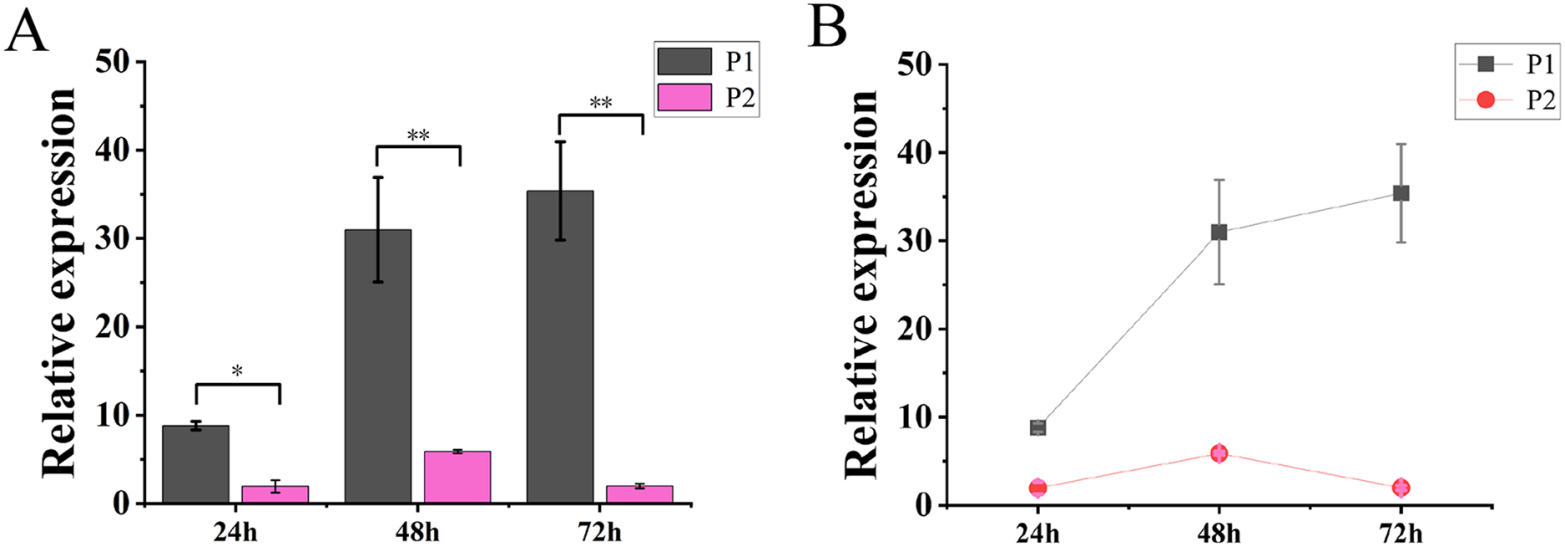
Expression levels of *TaGRF2-7A* at different time points after transformation. (A) Histogram of expression levels of T*aGRF2-7A* at different time points after transformation. (B) Line graph of expression levels of *TaGRF2-7A* at different time points after transformation. (P1, Tobacco transformed with pCAMBIA1304 -*35S::TaGRF2-7A* alone, P2, co-transformed with pCAMBIA1304-*35S::pre-miR396b* and pCAMBIA1304-*35S::TaGRF2-7A*)**. **Vertical bars represent standard deviation. Asterisks indicate significant difference or highly significant difference between P1 and P2. An asterisk (*) and two asterisks (**) indicate significant difference (*P* < 0.05) and highly significant difference (*P* < 0.01) using Student’s *t*-test, respectively.

The results of molecular reaction assay in tobacco showed that the expression level of *TaGRF2-7A* was continuously increased after transformation only with pCAMBIA1304-*35S::TaGRF2-7A*. When transformed with vector pCAMBIA1304 alone (P3), the transcripts of *TaGRF2-7A* was not detected. However, the expression level of *TaGRF2-7A* was very low when co-transformed with pCAMBIA1304-*35S::pre-miR396b* and pCAMBIA1304-*35S::TaGRF2-7A* (Fig. 12). This result demonstrated that *tae-miR396b* could interact with the transcripts of *TaGRF2-7A*, and negatively regulated the expression of *TaGRF2-7A*.

### The regulatory effect of *tae-miR396b* on *TaGRF2-7A* analyzed by GUS staining

**Figure 13:**
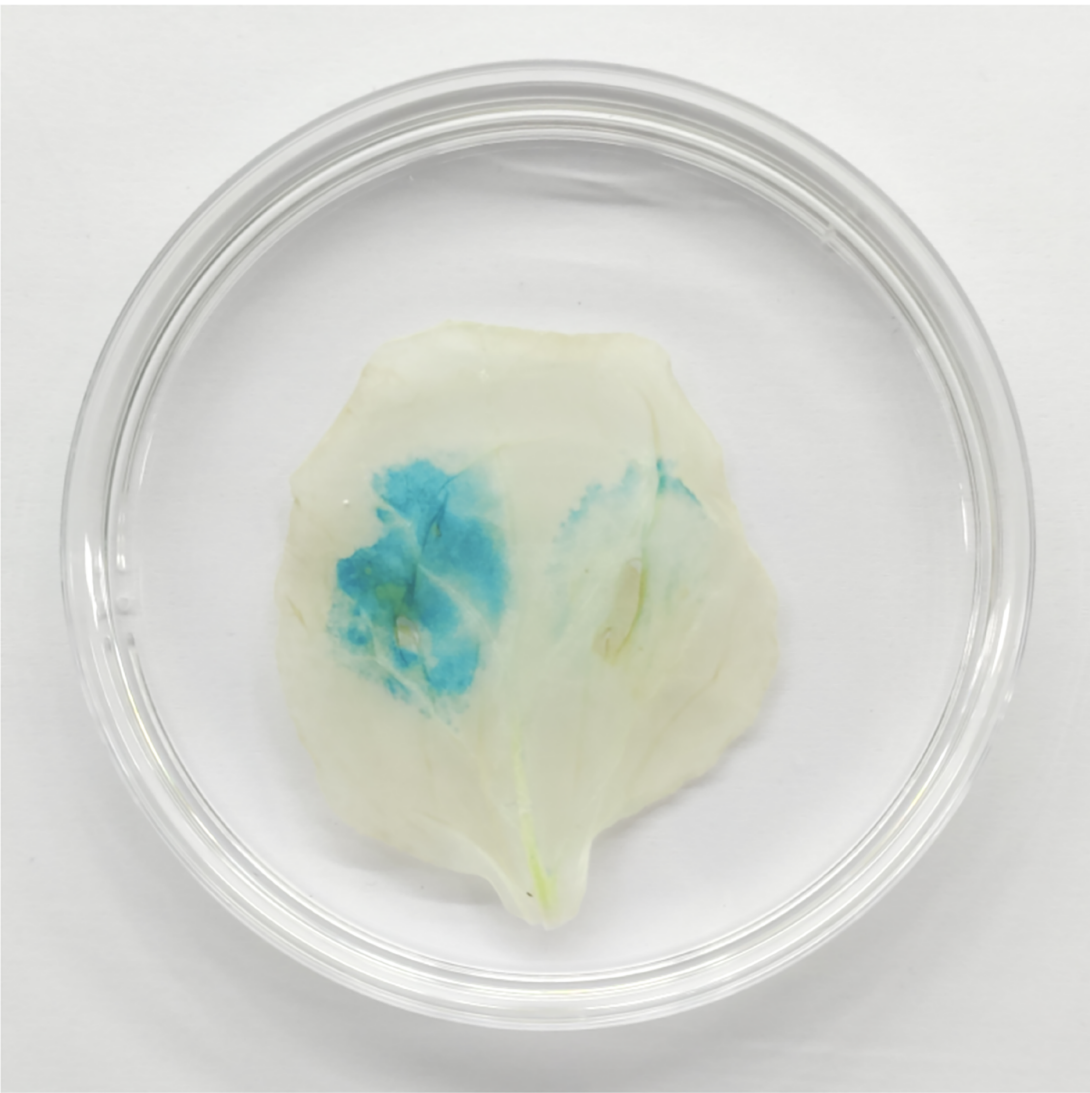
*TaGRF2-7A* regulated by *tae-miR396b* was verified by GUS chemical staining in tobacco leaf. Left side of tobacco leaf: transformed with pCAMBIA1304-35S::*TaGRF2-7A* alone; Right side of tobacco leaf: co-transformed with pCAMBIA1304-35S::pre-*miR396b* and pCAMBIA1304-35S::*TaGRF2-7A*.

GUS staining result indicated that the tobacco leaf transformed with only pCAMBIA1304-*35S::TaGRF2-7A* was stained deeply, indicating accumulation of the transcripts of *TaGRF2-7A*. Oppositely, the tobacco leaf transformed with both pCAMBIA1304-*35S::TaGRF2-7A* and pCAMBIA1304-*35S::pre-miR396b* could not be stained, indicating the transcripts of *TaGRF2-7A* were digested (Fig. 13). This result demonstrated that *TaGRF2-7A* was a target of *tae-miR396b*. The expression of *TaGRF2-7A* was negatively regulated by *tae-miR396b*.

## Discussion

### Characteristics and functions of *tae-miR396b* and *TaGRFs*

*Tae-miR396b* has three homeologous genes located on the long arms of chromosomes 6A, 6B, and 6D, with some SNPs and Indels among them (Fig. 3B). SNP markers obtained from molecular genetics and genomics provide broad prospects for wheat breeding (Collard & Mackill, 2007). The SNPs and Indels can be used to distinguish the three homeologous genes of *tae-miR396b*, and used in wheat molecular design breeding.

Plant GRF proteins typically have two conserved domains at the N-terminus: QLQ and WRC. QLQ domains interact with GRF interactors (GIFs) to form transcriptional activators involved in biological processes of plant growth and development (Kim & Kende, 2004). The expression levels of *GRFs* are usually higher in actively growing tissues than in mature tissues (Horiguchi, Kim & Tsukaya, 2005; Kim, Choi & Kende, 2003; Rodriguez et al., 2010). Wheat *GRF* gene family have 30 members. *TaGRFs* have a large number of *cis*-regulatory elements related to growth and development, hormones and stress (Zhang et al., 2021). Gene ontology annotation showed that *TaGRFs* were mainly involved in ATP binding (GO:0005524), regulation of DNA templated transcription (GO: 0006355) and developmental processes (GO:0032502) (Table 2). These indicated that *TaGRFs* also played important roles in wheat development. A total of 23 *TaGRFs* had the target sites of *tae-miR396b* (Fig. 4), indicating their expressions were regulated by *tae-miR396b*. These suggested that *tae-miR396b* and *TaGRFs* played important roles in wheat growth and development.

### *Tae-miR396b* and *TaGRFs* were involved in the regulation of wheat spike development

More and more studies have shown that *miR396* is involved in developments of plant leaves, roots, florets and grains by regulating *GRFs* (Bazin et al., 2013; Liu et al., 2009; Liu et al., 2014; Yu et al., 2020). The regulation mechanism of *miR396* on crop growth is complex, and it can offset the interference caused by external changes through autonomous regulation. The *miR396*-mediated *GRF* regulation (*miR396*-*GRF*) module has been demonstrated as a potential application in improving plant biomass, crop yield, stress tolerance, and increasing the efficiency of genetic transformation in plants (Cao et al., 2007; Chen et al., 2019; Kong et al., 2020). Overexpression of *miR396*-resistant *GRF1* do not increase leaf size, because *miR396a* can control maize leaf growth by finely controlling transcript levels through a series of complex processes to counteract the effects of overexpression (Nelissen et al., 2015). Tissue specific expression analysis of *TaGRFs* indicated that the most *TaGRFs* were expressed in both roots and leaves, but all *TaGRFs* expressed at high levels in young spikes (Fig. 5). The results of qRT-PCR showed that the expression levels of *TaGRFs* were varied at different spike developmental stages (Figs. 6 and 7). These suggested that *TaGRFs* were involved in the regulation of wheat spike development.

### *Tae-miR396b* and *TaGRFs* were involved in the abnormal spike differentiation of mutants *drs*, *ass*, and *ptsd1*

*GRFs* are involved in the formation of the reproductive organs of *A. thaliana*, affecting the differentiation of female and stamen, and the formation of embryo sac. It is of great significance for the reproductive capacity and generational continuity of *A. thaliana* (Lee et al., 2014). In maize, 14 *ZmGRF* genes have been identified, *ZmGRF2* and *ZmGRF11* may be involved in the growth and development of spike formation and contribute to the improvement of plant yield (Zhang et al., 2008). Rice *GRFs* are involved in floral organ development (Li et al., 2010; Luo et al., 2005); overexpression of *OsmiR396d* targeted inhibition of *OsGRF6* leads to dysplasia of flower organs (Liu et al., 2014); both *OsGRF6* and *OsGRF10* can bind the promoters of *OsCR4* and *OsJMJ706*, which influence rice fertility and floral organ development (Pu et al., 2012; Sun & Zhou, 2008). The mutants had the same genetic background with WT. Genetic study demonstrated that a single or two Mendelian genes controlled the spike phenotypes of the mutants. The expression levels of *tae-miR396b* and *TaGRFs* in spikes of WT and mutants *drs*, *ass* and *ptsd1* at different stages were significantly different (Figs. 6 and 7). The most significantly differentially expressed genes were *TaGRF1-6A*, *TaGRF2-7A*, *TaGRF4 (6A, 6B, 6D)*, *TaGRF5-7A*, *TaGRF6 (4A, 4D*) and *TaGRF9 (4A, 4D)*. In *Arabidopsis*, lines with *GRF3* mutations in the *miR396* binding site and lines overexpressing *AtGRF5* can effectively delay leaf senescence (Debernardi et al., 2014). *GRFs* are also involved in the growth and development of plant roots, stems (Kim, Choi & Kende, 2003), and flowers (Liang et al., 2013). We speculated that these *TaGRFs* with significantly different expression levels played important roles in spike development. The result suggested that the *tae-miR396b-TaGRFs* module was involved in the abnormal spike development of the mutants. Correlation analysis showed that *tae-miR396b* was positively or negatively correlated with *TaGRFs* (Figs. 8–11). The positive correlations were contradictive with the regulatory relationships predicted between *Tae-miR396b* and *TaGRFs*. This result suggested that the expressions of *TaGRFs* were regulated by a complex system, not only by *tae-miR396b*. The expression of *TaGRF2-7A* could be inhibited by *tae-miR396b* directly (Figs. 12 and 13), but whether all the other *TaGRFs* were regulated directly by *tae-miR396b* still needed to be verified.

## Conclusions

Three homeologous genes of *tae-miR396b* are on the long arms of chromosome 6A, 6B and 6D with some SNPs and Indels. A total of 23 *TaGRFs* have the target sites of *tae-miR396b*, and they express at the highest levels in wheat spikes. *Tae-miR396b* and *TaGRFs* express at significantly different levels in spikes of Guomai 301 and mutants *drs*, *ass* and p*tsd1* at the floret primordium visible stage, the female and male primordium differentiation stage, and the green anther stage. The most *TaGRFs* are negatively regulated by *tae-miR396b*, which have different regulatory effects on *TaGRFs* in WT and the three spike mutants. The expression of *TaGRF2-7A* is directly negatively regulated by *tae-miR396b*. The expressions of *TaGRFs* are regulated by a complex system, not only by *tae-miR396b*. *Tae-miR396b* and *TaGRFs* are involved in the regulation of wheat spike development, and play important roles in the abnormal spike development of the mutants. This study has identified *tae*-mi*R*396 and its important potential target *GRF* family gene members, revealed the roles of *tae-miR396b-TaGRFs* module in spike differentiation of wheat, and laid a foundation for further research on the roles of *tae-miR396b* and *TaGRFs* in spike development of wheat. It has provided candidate functional genes for wheat breeding, especially for spike improvement.

## Supplemental Information

10.7717/peerj.18550/supp-1 Supplemental Information 1 The DNA sequences of the primers used for genes expression analysis.

10.7717/peerj.18550/supp-2 Supplemental Information 2 Target gene prediction results of tae-miR396b.

10.7717/peerj.18550/supp-3 Supplemental Information 3 The transcripts per million (TPM) values of the *TaGRFs* in roots, leaves and spikes of Chinese Spring at vegetative and reproductive stages.

10.7717/peerj.18550/supp-4 Supplemental Information 4 The raw data of qRT-PCR.

## Additional Information and Declarations

### Data Availability

The following information was supplied regarding data availability: The raw data of qRT-PCR are available in the Supplemental Files.
