# Supplementary material for: Tae-miR396b regulates TaGRFs in spikes of three wheat spike mutants
Source: PeerJ. 2024 Nov 22;12:e18550. doi: 10.7717/peerj.18550 (PMC11587873; doi:10.7717/peerj.18550)
Supplement: Supplemental Information 1 [file peerj-12-18550-s001.docx]

**Table S1** **The DNA sequences of the primers used for genes expression analysis**

|  | Gene name | Forward primer (5′-3′) | Reverse primer (5′-3′) |
| --- | --- | --- | --- |
| 1 | *TaGRF1-6A* | ATCTCCATCGCCAATGAACTC | CTTCGTTCTTGGTCTTCTCGTT |
| 2 | *TaGRF1-6B* | GCTTTGGTCGAGTGTAACATTT | CGGAAGCTAGCATCTCATCTAA |
| 3 | *TaGRF1-6D* | CCAGCTAGCTATCTTGACTCTC | AAATGTTACACTCGACCAAAGC |
| 4 | *TaGRF2-7A* | GTTCAAGAAAGCCTGTGGAAC | TTTATCTGGCTCTGAGAAGCAG |
| 5 | *TaGRF2-7B* | GGGTAGGCCCTGGAATATGG | ATTCATCGTTGTGGTGGTGGT |
| 6 | *TaGRF2-7D* | CTGTGGAACCGATGTCCTCCTC | GGTGGAGCTGGAGAGGGTTTAT |
| 7 | *TaGRF3-2A* | CATGACTCCAACCACAACAATG | CACCTTCACCGGGAGTATATAC |
| 8 | *TaGRF3-2D* | ATTCTGCCTATGGGTTTAGGAC | GGTAGCTCGAGAGTGAGAAC |
| 9 | *TaGRF4-2B* | ATTCTGCCTATGGGTTTAGGAC | GAGTGAGAAGGATGAGTTCTGG |
| 10 | *TaGRF4-6A* | CTTCTCAAACGACTACATTCCC | TTGTTGTTGCTGCTGTTGTTC |
| 11 | *TaGRF4-6B* | GTTCAAGAAAGCCTGTGGAAAC | TAGAGGGAGTGGTTGTGGAAG |
| 12 | *TaGRF4-6D* | GACCTCATTCCCGCTTTCAAG | CTGTTGTTGTGGTTGTTGTTCT |
| 13 | *TaGRF5-4A* | GCTTCACCATCGATTCCCCA | GCTGGTTCTCCTGCTTGACA |
| 14 | *TaGRF5-7A* | GTCATACGGCAACAAATACTCC | CTGTTGTTGTTTCCTGATGCTC |
| 15 | *TaGRF5-7D* | ATACGGCGGCAAATACTCCC | CAGTTGGTATGGGGTCAGGC |
| 16 | *TaGRF6-4A* | CCCGCTTCTTGCTTAGGGTT | CCCAATGTGGAGGAGCCAAA |
| 17 | *TaGRF6-4B* | CATTGATCCTTGTTCACTGCAA | GTGATTGTGATGCTCCTTTGAG |
| 18 | *TaGRF6-4D* | CAACAAACACCAACAGGACTC | GGAGGAAGCATCTCTATTCCTC |
| 19 | *TaGRF9-4A* | GTCCAATGAGCAGAGGCTGT | GGCCTCCAGGGCATTCTATC |
| 20 | *TaGRF9-4D* | ATGCCCTTCACTCCTTCACAG | TCTGCCGTTCTTTACCGC |
| 21 | *TaGRF10-6A* | GTCAGTTTGACAGTTACCGTTG | CTATGGAGGGAGAGTGTTCTTG |
| 22 | *TaGRF10-6B* | GAGCCTGTGCTTCGACTACC | CCACAGGCTTTCTTGAACGG |
| 23 | *TaGRF10-6D* | GAGCCTGTGCTTCGACTACC | CCACAGGCTTTCTTGAACGG |
| 24 | *Actin* | GCCATCCAAGCTGTTCTCTC | GCTCGTAGTCAACAGCAACAA |
| 25 | *U6* | GCTCGTAGTCAACAGCAACAA | ATTTGGACCATTTCTCGATTTGTGC |
| 26 | *Tae-miR396b* | GCGTTCCACAGCTTTCTTGAACTT |  |
